# Supplementary material for: Chemo-mechanical forces modulate the topology dynamics of mesoscale DNA assemblies
Source: Nat Commun. 2023 Oct 13;14:6459. doi: 10.1038/s41467-023-41604-z (PMC10575982; doi:10.1038/s41467-023-41604-z)
Supplement: Supplementary file 1 — Supplementary Information [file 41467_2023_41604_MOESM1_ESM.pdf]

Supplementary Information

Chemo-mechanical forces modulate the topology dynamics of mesoscale DNA assemblies

Deepak Karna[1], Eriko Mano[2], Jiahao Ji[1], Ibuki Kawamata[3,*], Yuki Suzuki[2,4,*], Hanbin Mao[1,*]

[1]Department of Chemistry and Biochemistry, Kent State University, Kent, Ohio, 44242, USA

[2]Frontier Research Institute for Interdisciplinary Sciences, Tohoku University, 6-3 Aramaki-aza Aoba, Aoba-ku, Sendai, 980-8578, Japan

[3]Department of Robotics, Graduate School of Engineering, Tohoku University, 6-6-01 Aramaki-aza Aoba, Aoba-ku, Sendai, 980-8579, Japan

[4]Department of Chemistry for Materials, Graduate School of Engineering, Mie University, Tsu 514-8507, Japan

Corresponding Authors:
IK, ibuki.kawamata@tohoku.ac.jp, YS, ysuzuki@chem.mie-u.ac.jp, and HM, hmao@kent.edu

Contents

Suppl. Fig. 1. Schematic of nanospring indicating each transformable module unit.................................. 3

Suppl. Fig. 2. Detailed structure of nanospring with each DNA sequences 4

Suppl. Fig. 3. Representative atomic force microscopy (AFM) images of GQ-NS and anti-GQ-NS under different oligo conditions. 6

Suppl. Fig. 4. Representative atomic force microscopy (AFM) images of GQ-NS and anti-GQ-NS under different buffer conditions. 8

Suppl. Fig. 5. Analysis of curvatures from AFM images of GQ-NS.. 9

Suppl. Fig. 6. Histograms for the measurement of contour length of nanosprings................................... 10

Suppl. Fig. 7. Fitting of GQ-NS and anti-GQ-NS. 11

Suppl. Fig. 8. Temporal traces of forces and extension during force jump events for anti-GQ-NS 12

Suppl. Fig. 9. Overlapping stretching traces in the same nanospring construct 13

Suppl. Fig. 10. Subsequent sets of force-jump experiments carried out for same GQ-NS molecule 14

Suppl. Fig. 11. Representative high-resolution AFM images of GQ-NS 15

Suppl. Fig. 12. Representative high-resolution AFM images of anti-GQ-NS 16

Suppl. Fig. 13. Different AFM scanning directions for anti-GQ-NS and GQ-NS..................................... 17

Suppl. Fig. 14. Coarse-grained simulation with 21 bp linkers.. 18

Suppl. Fig. 15. Coarse-grained simulation with 4 nt linkers... 19

Suppl. Fig. 16. Simulation results fitted to form helix showing radius and pitch...................................... 20

Suppl. Fig. 17. Bar plots to show the thermal fluctuations have non-negligible effect on pitch of helix ... 21

Suppl. Fig. 18. Coarse-grained simulation with no linkers.. 22

Suppl. Fig. 19. Coarse-grained simulation with single-stranded 21 nt linker 23

Suppl. Note: Specification of the radius and pitch of a helix.. 24

Suppl. Fig. 1. Schematic of nanospring indicating each transformable module unit

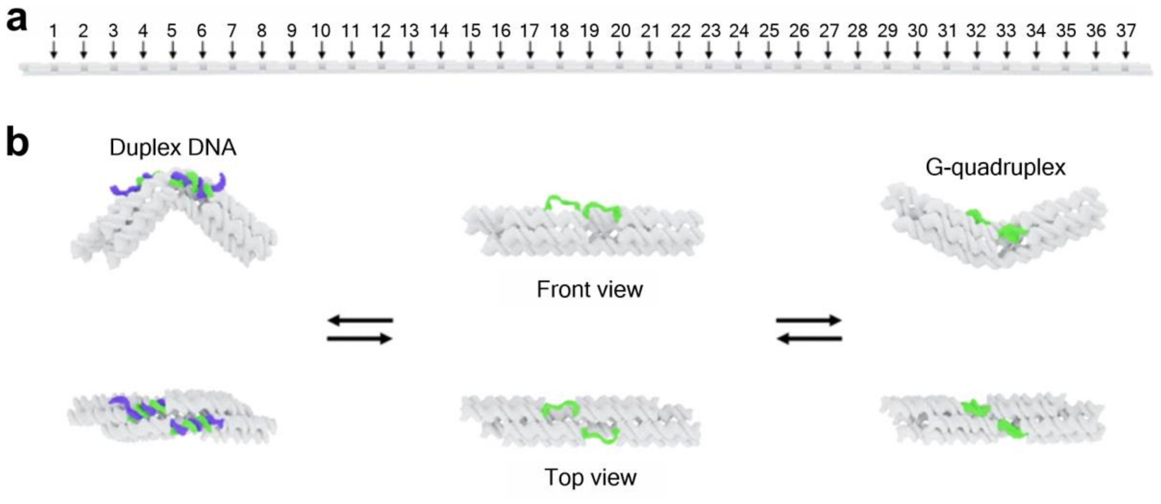

Suppl. Fig. 1. Schematic of nanospring that shows positions of transformable module unit within the origami. a A model of linear nanospring with numbered arrows pointing to positions of several transformable modules. **b** Each module comprises of bridging strands (either duplex DNA or G-quadruplex). Note that bending directions change as per the effect of specific bridging strands.

Suppl. Fig. 2. Detailed structure of nanospring with each DNA sequences

See next page for this figure. Please enlarge to see the details.

Suppl. Fig. 2. General structure with detailed sequence information for the DNA origami nanospring. The six helices are numbered from 0 to 5 represented in circles. The continuous blue line represents the p8064 plasmid while grey lines denote several staples.

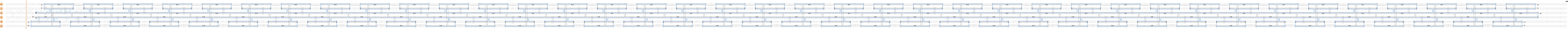
0
1
2
3
4
5

Suppl. Fig. 3. Representative atomic force microscopy (AFM) images of GQ-NS and anti-GQ-NS under different oligo conditions.

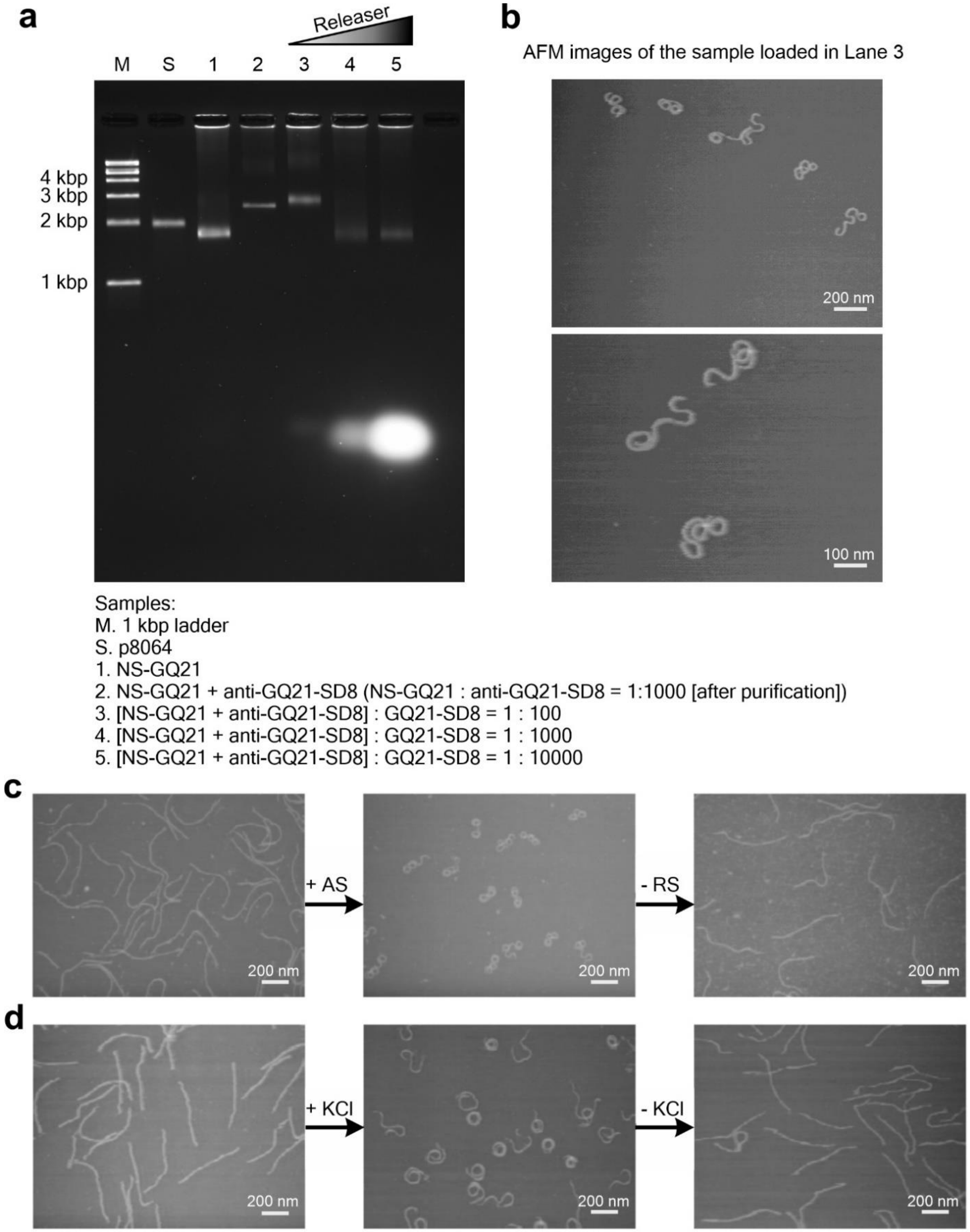

Suppl. Fig. 3: Agarose gel electrophoresis (AGE) and AFM analyses of reversible switching of the nanospring shape. **a** AGE analysis. To make the anti-GQ nanospring (anti-GQ-NS), anti-GQ strands (ASs) were first added at the molar ratio of nanospring: anti-GQ strand = 1 : 1000. The excess volume of unincorporated anti-GQ strands was then removed by the PEG-sedimentation-based purification. After that, releaser strands (RSs) were added at the molar ratio of nanospring: releaser strand = 1: 100, 1: 1000, or 1: 10000. M: 1 kbp ladder marker; S: p8064 scaffold; lane 1: NS before incubation with AS; lane 2: NS after incubation with anti-GQ strand AS (anti-GQ-NS); lanes 3–5: anti-GQ-NS after incubation with different concentrations of releaser strand. Anti-GQ-NS: releaser = 1:100 (lane 3), 1:1000 (lane 4), and 1:10000 (lane 5). Excess amount of RSs were observed as the lower bands in lanes 3-5. The higher band observed in lane 4 may reflect the formation of an intermediate complex in which both an anti-GQ and releaser strands remain bound to an GQ-forming strand. **b** AFM images of sample loaded into Lane 3 of Suppl. Fig. 3a. **c** Reversible switching of the nanospring shape via toehold-mediated strand displacement reaction. Representative AFM images of nanospring of two independent experiments before and after incubation with AS and after subsequent incubation with RS are shown. **d** Reversible switching of the nanospring shape *via* folding/unfolding of G-quadruplex. Representative AFM images of nanospring of two independent experiments before and after incubation with 100 mM KCl and after subsequent removal of KCl from the sample are shown. The reversible transformation of the construct with GQ-forming sequences was achieved by changing the KCl concentration in the buffer from 0 mM and 100 mM. A 100 kDa MWCO centrifuge filter (Amicon Ultra, Merck Millipore, Billerica) was used to exchange the buffer.

Suppl. Fig. 4. Representative atomic force microscopy (AFM) images of GQ-NS and anti-GQ-NS under different buffer conditions.

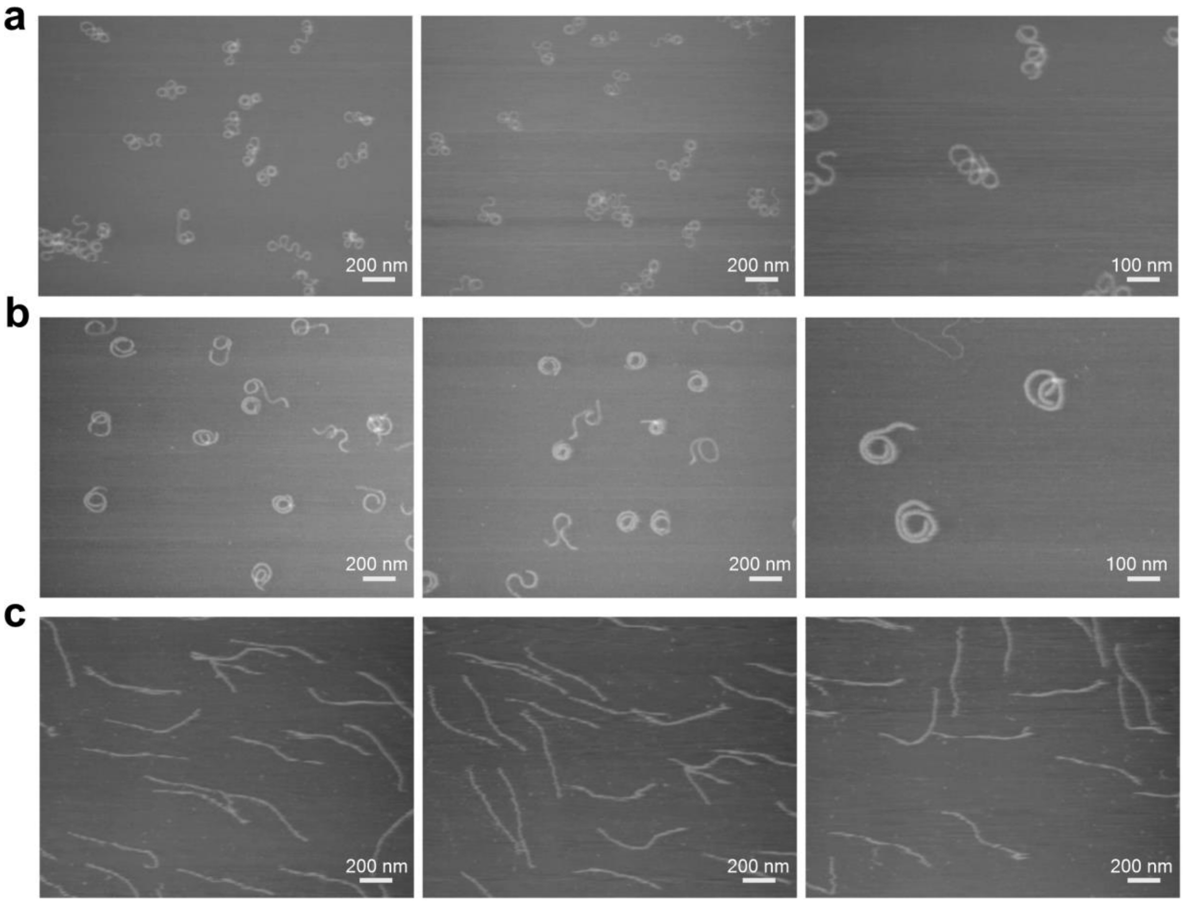

Suppl. Fig. 4: Representative AFM images of nanosprings. a Anti-strand-incorporated nanospring (anti-GQ-NS). Representative images of over three independent experiments. **b** G-quadruplex-induced nanosprings (GQ-NS). Representative images of over three independent experiments. **c** Nanospring (GQ-NS) in the presence of 100 mM of LiCl. Representative images of over two independent experiments.

Suppl. Fig. 5. Analysis of curvatures from AFM images of GQ-NS

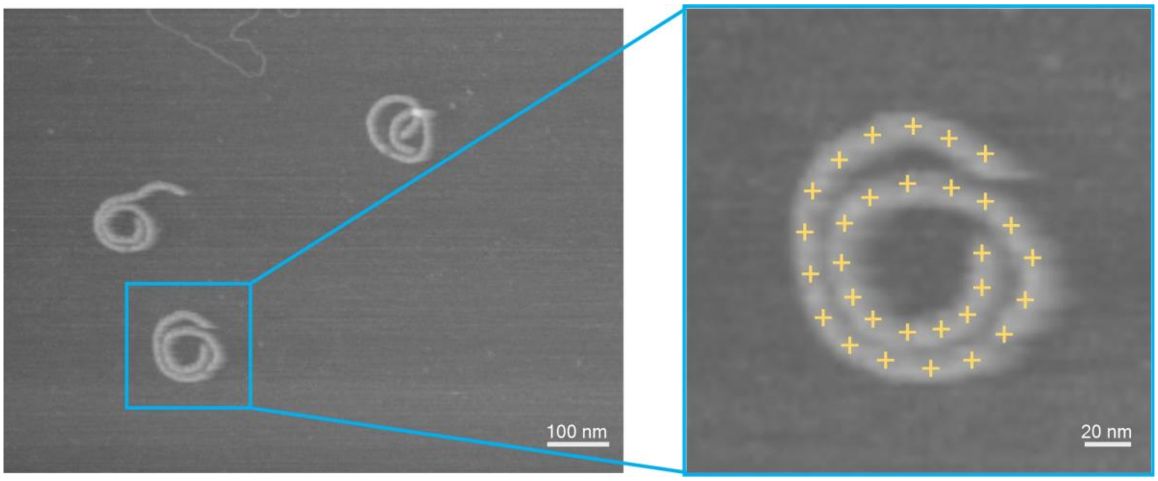

Suppl. Fig. 5: Analysis of AFM images. Thirty points having coordinates (x_1, y_1), (x_2, y_2), ..., (x_i, y_i) ..., (x_{30}, y_{30}) were taken along the nanospring. The distance along the contour L_i and cosθ_i for each i (1≦i≦30) 30 were then calculated. θ_i of cosθ_i is the angle of vectors (x_2-x_1, y_2-y_1) and (x_{i+1}-x_i, y_{i+1}-y_i), which is computed using inner product of vectors. (Thus, θ_1=0 and cosθ_1=1. θ_{30} is undefined.) L_i was calculated as $\sum_{k=2}^{i} \| (x_k - x_{k-1}, y_k - y_{k-1}) \|$, where $\|\mathbf{v}\|$ is the distance (or norm) of the vector **v**. Thus, L_{30} approximates the contour length of the DNA structure. L_1 is defined L_1=0. To compute the average curvature, the fitted circle was first obtained from six consecutive points for every i from 1 to 25 using least squares method. Then, local curvatures were calculated as a reciprocal of the radius of the circles. Finally. we obtained the average curvature by averaging the values from all i = 1 to 25.

Suppl. Fig. 6. Histograms for the measurement of contour length of nanosprings.

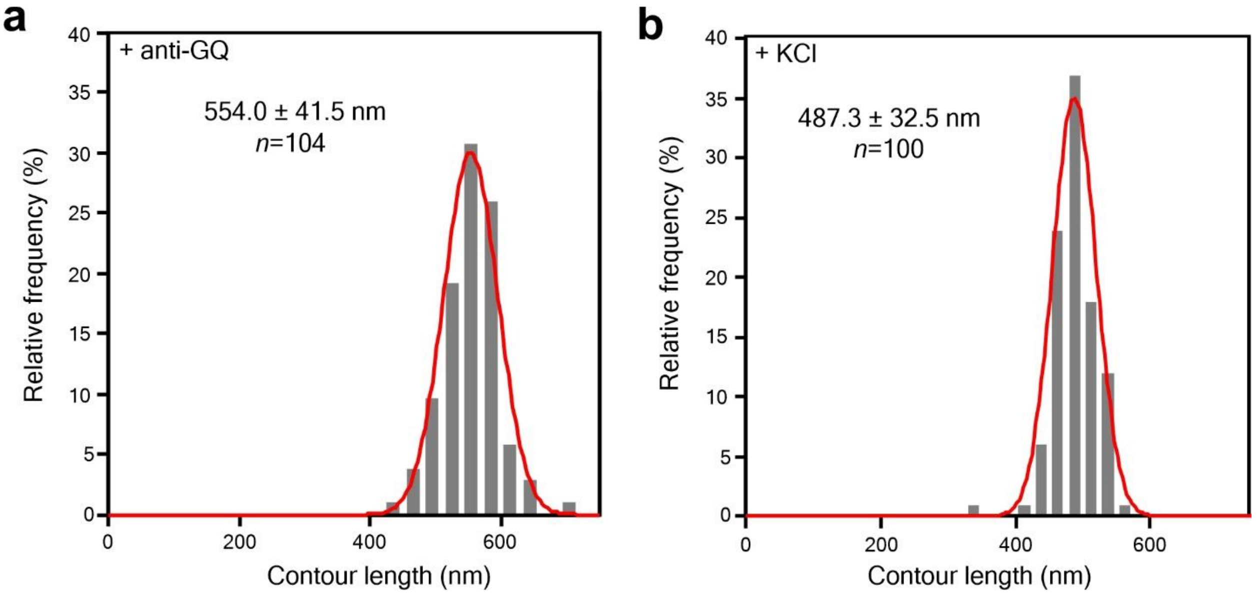

Suppl. Fig. 6: Histograms for the analysis of the contour length a anti-GQ-NS **b** GQ-NS. The data have been collected by estimation from the apparent shape using 30 points along the shape (see Suppl. Fig. 5).

Suppl. Fig. 7. Fitting of GQ-NS and anti-GQ-NS.

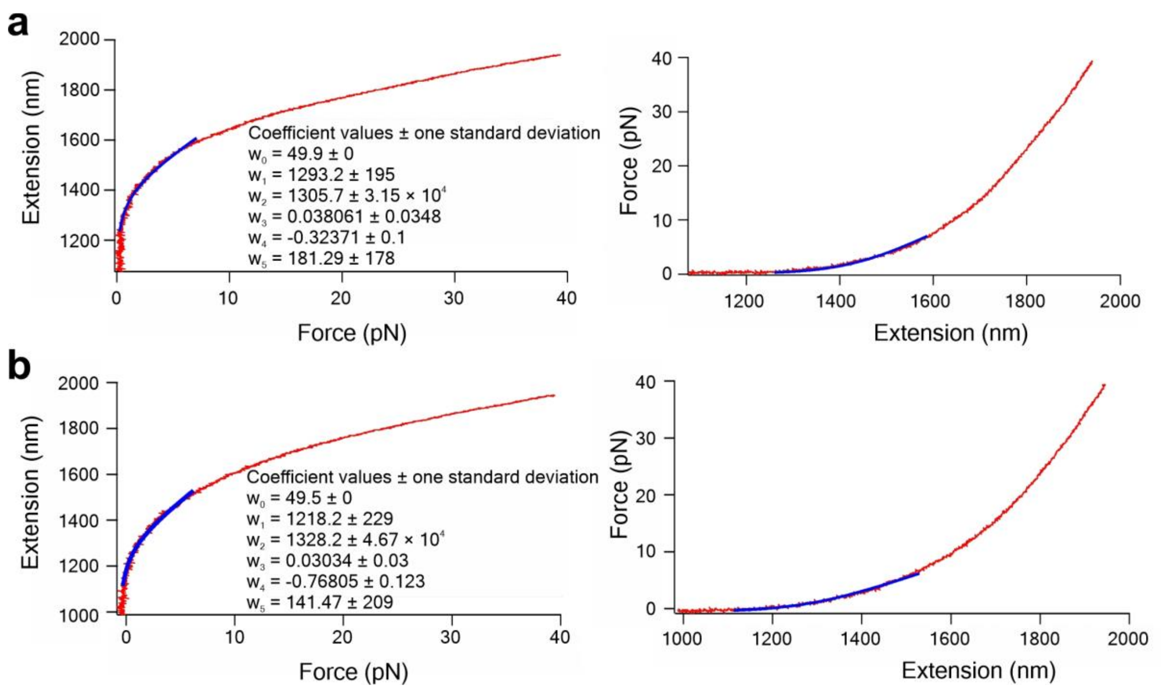

Suppl. Fig. 7: Fitting equation to solve for length of nanospring at ≈0 to 8 pN. **a** Fitting parameters obtained for GQ-NS from force-extension curve. **b** Fitting parameters obtained for anti-GQ-NS from force-extension curve. Red trace indicates the force-extension curve while blue trace represents the fitted curve. Fitting model and equations are discussed in Methods section.

Suppl. Fig. 8. Temporal traces of forces and extension during force jump events for anti-GQ-NS

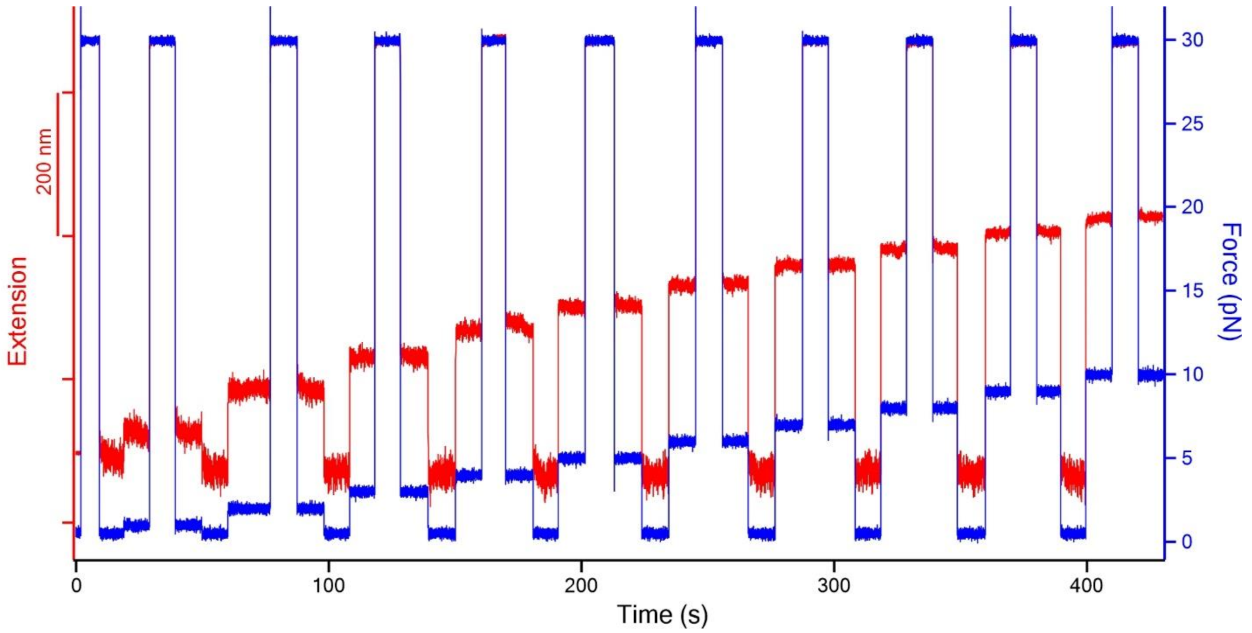

Suppl. Fig. 8: Temporal traces of forces and extensions during different force-jump events in anti-GQ nanospring. Red trace represents the extension corresponding to respective force (marked as blue trace) over a period of time (shown in x-axis).

Suppl. Fig. 9. Overlapping stretching traces in the same nanospring construct

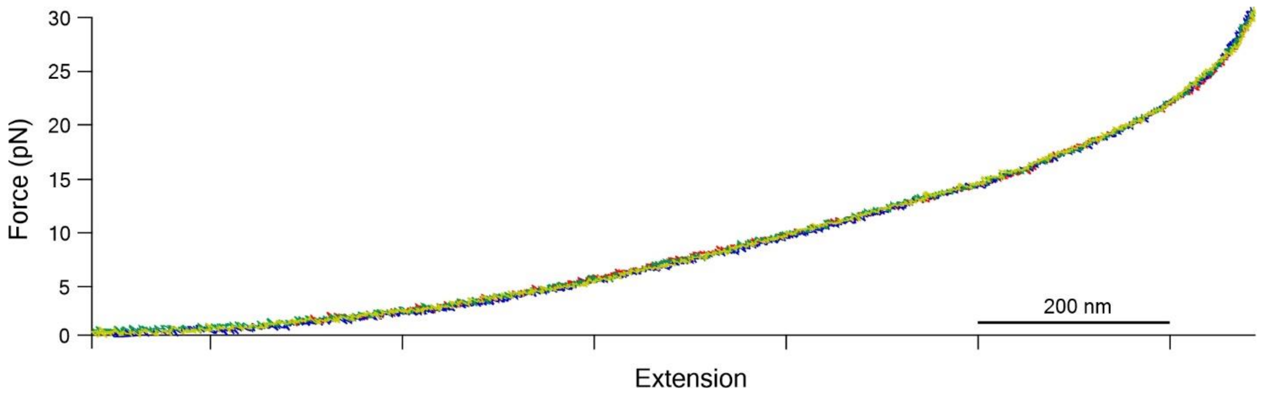

Suppl. Fig. 9: Multiple overlapped force-extension stretching curves for the same anti-GQ-nanospring. Subsequent traces following the same path during stretching of the nanospring up to 30 pN force indicate no structural deformations of the nanospring occurred during repetitive force-ramping processes. Different colors represent subsequent force-extension traces for the same molecule.

Suppl. Fig. 10. Subsequent sets of force-jump experiments carried out for same GQ-NS molecule

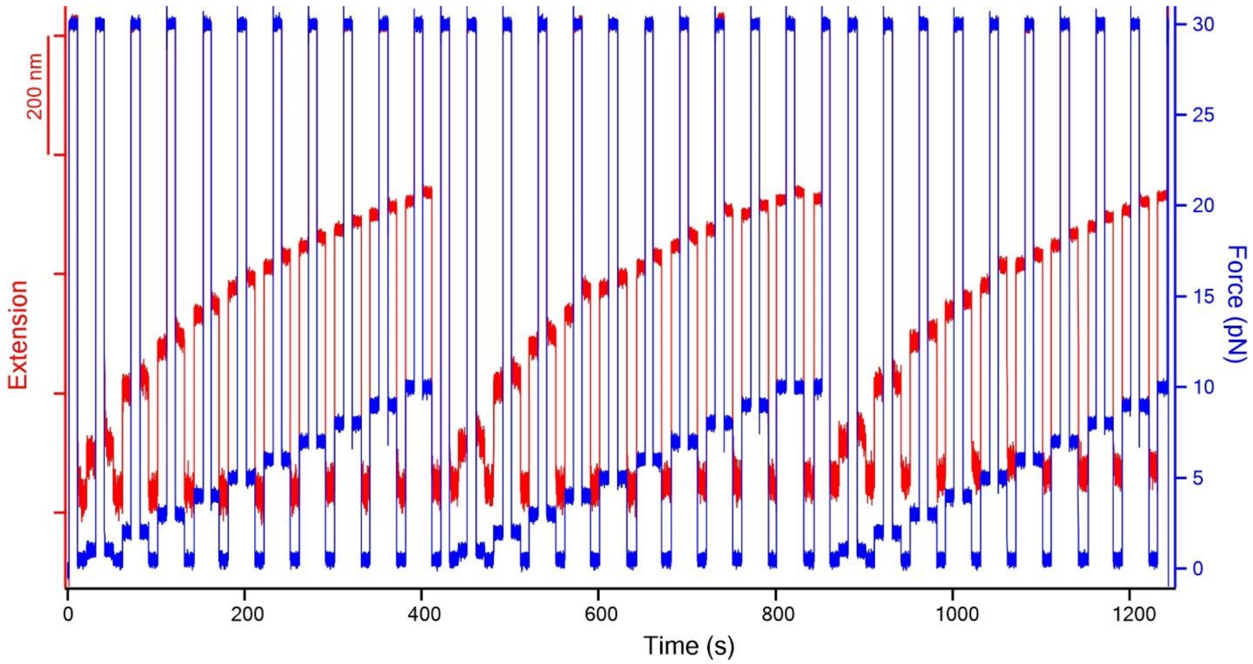

Suppl. Fig. 10: Three consecutive sets of force jump experiments carried out for the same GQ-nanospring. Each set consists of the force jump from 30 to 0.5, 1, 2 till 10 pN as well as from 0.5 pN to 1, 2, 3 till 10 pN.

Suppl. Fig. 11. Representative high-resolution AFM images of GQ-NS

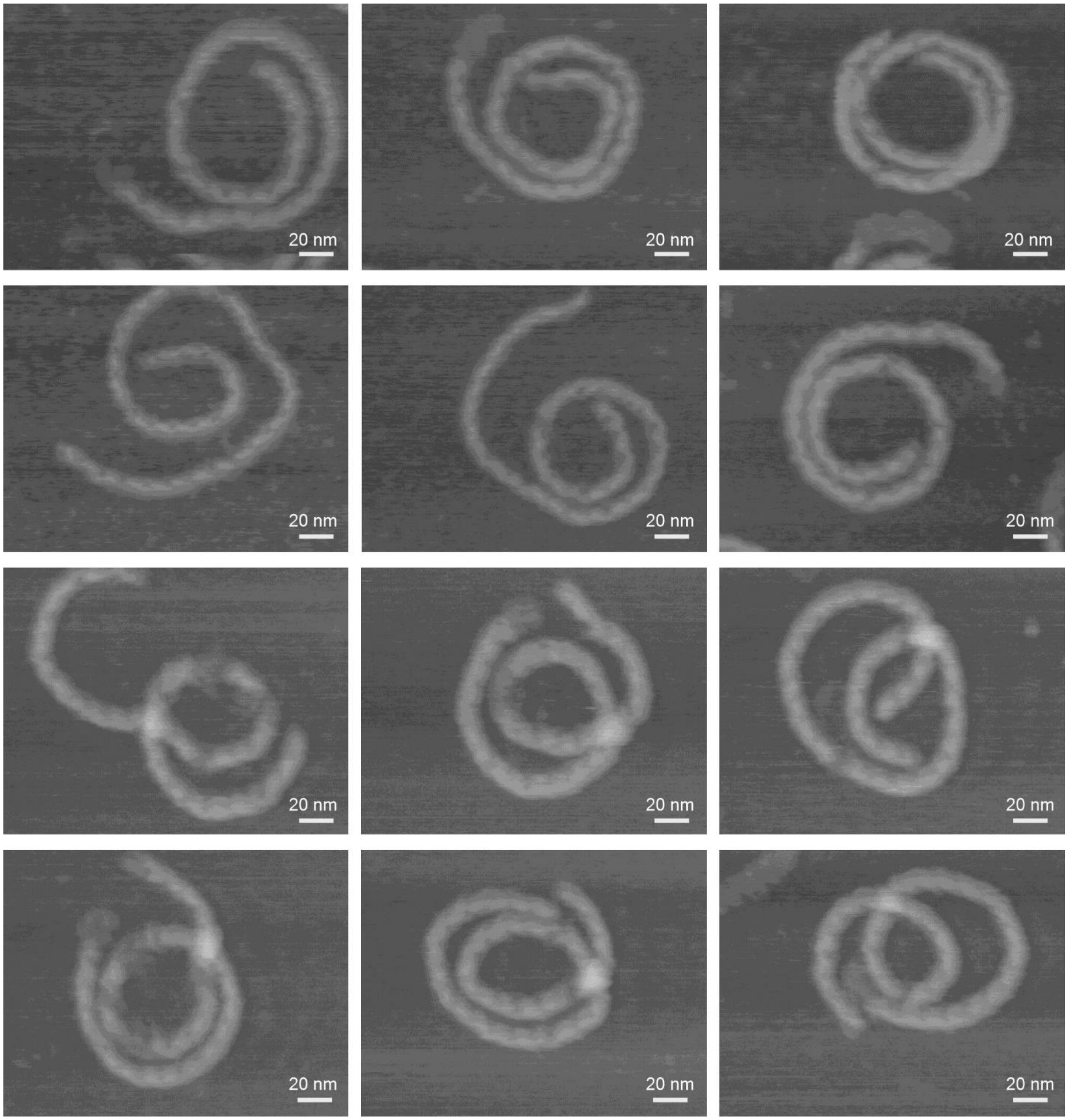

Suppl. Fig. 11: Representative high-resolution AFM images of GQ-NS. Note the slits (Fig. 1a for schematic) of each backbone in each figure are facing inwards. Representative of three independent experiments.

Suppl. Fig. 12. Representative high-resolution AFM images of anti-GQ-NS

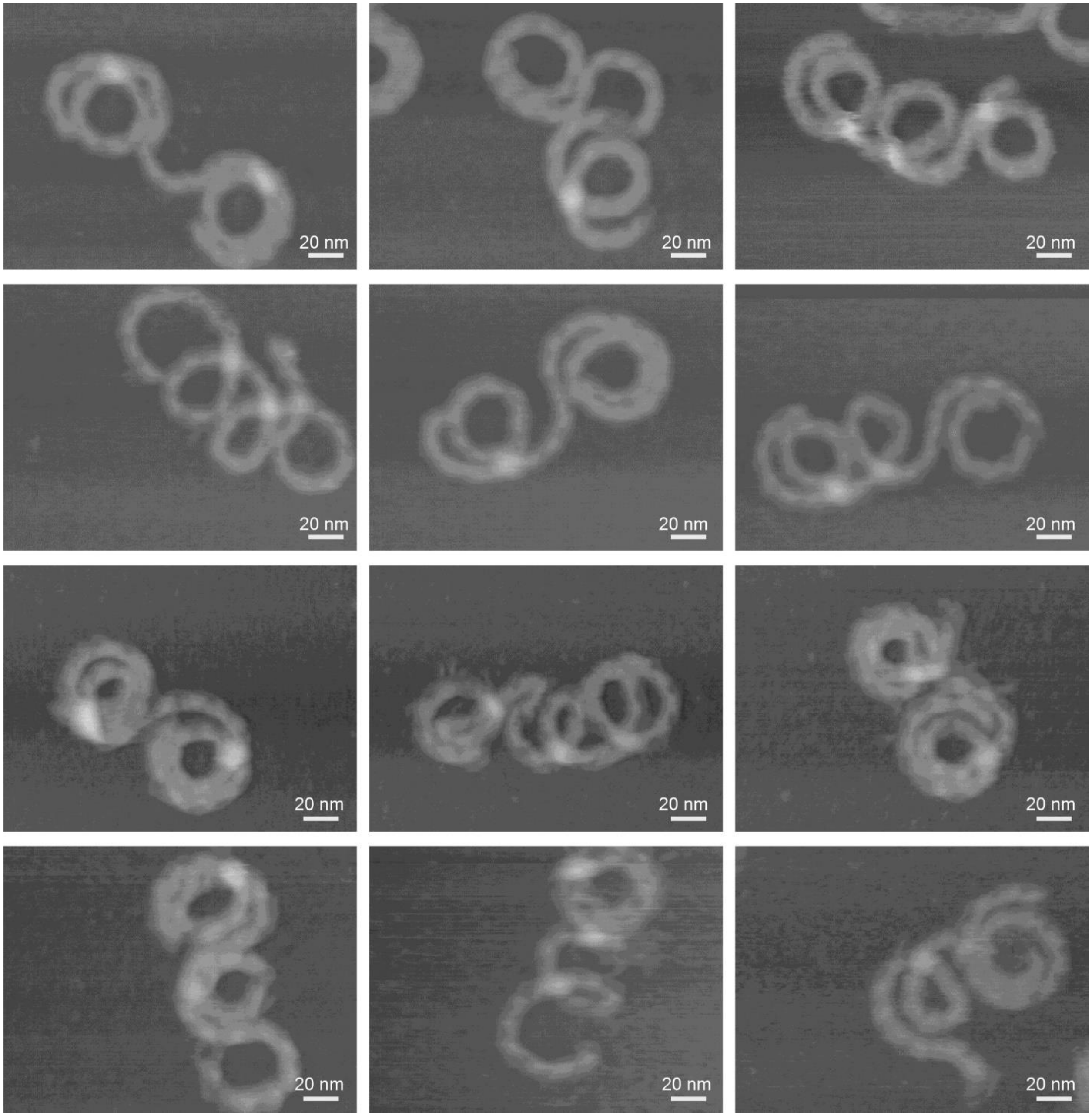

Suppl. Fig. 12: Representative high-resolution AFM images of anti-GQ-NS. Note the slits (Fig. 1a for schematic) of each backbone in each figure are facing outwards. Representative of four independent experiments.

Suppl. Fig. 13. Different AFM scanning directions for anti-GQ-NS and GQ-NS

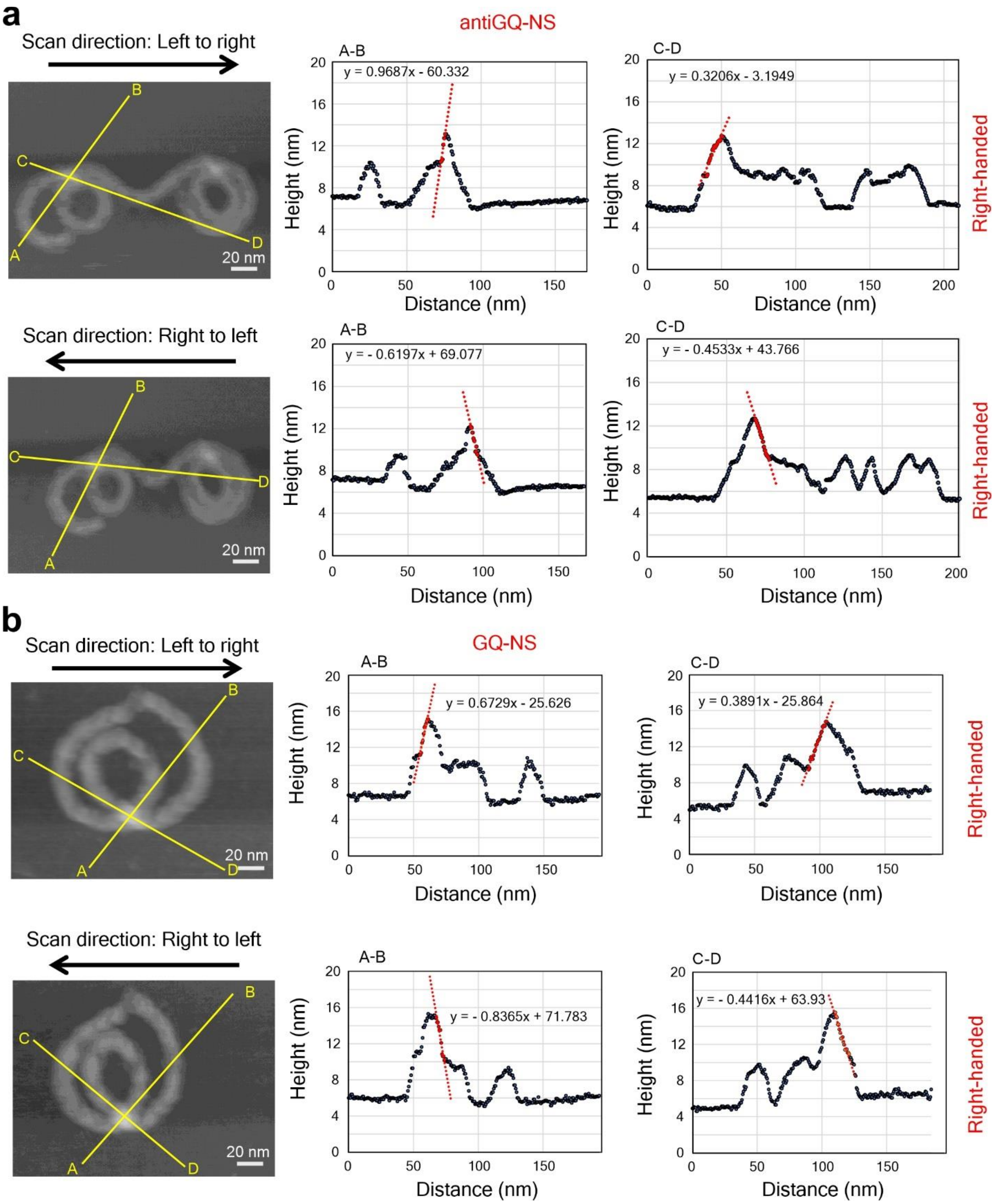

Suppl. Fig. 13: Different scanning directions of AFM. a anti-GQ-NS and **b** GQ-NS**.** Both scanning directions (either from left to right or from right to left) revealed right-handed chirality of nanosprings. Representative of two independent experiments.

Suppl. Fig. 14. Coarse-grained simulation with 21 bp linkers

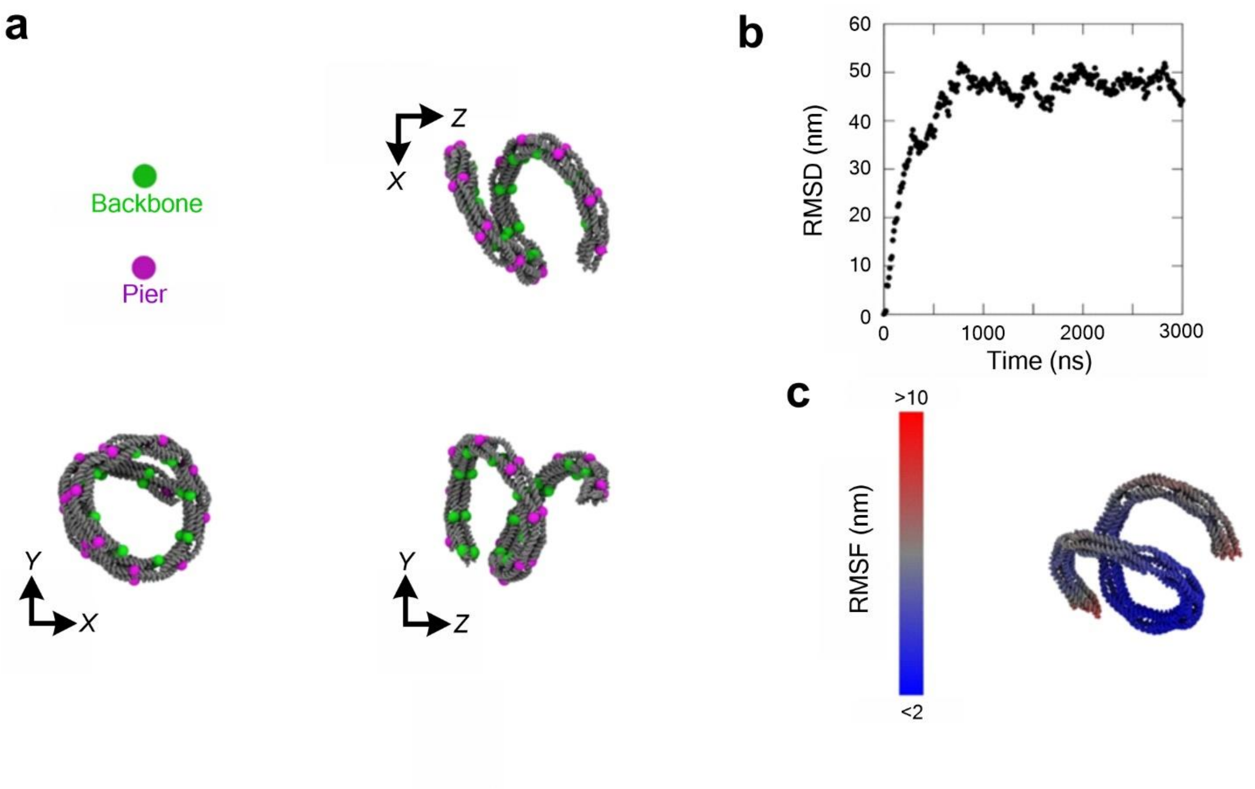

Suppl. Fig. 14: Detailed results of coarse-grained simulation where the 21 bp linkers were introduced as bridges. a Structure of the final frame at 3000 ns from orthographic views. Entities in backbone and pier are selected repeatedly and emphasized as green and magenta spheres, respectively. **b** Graph that shows the trajectory of RMSD **c** Graph that shows the RMSF in blue-to-red color scale, which is computed from 100 structures between 2000 to 3000 ns.

Suppl. Fig. 15. Coarse-grained simulation with 4 nt linkers

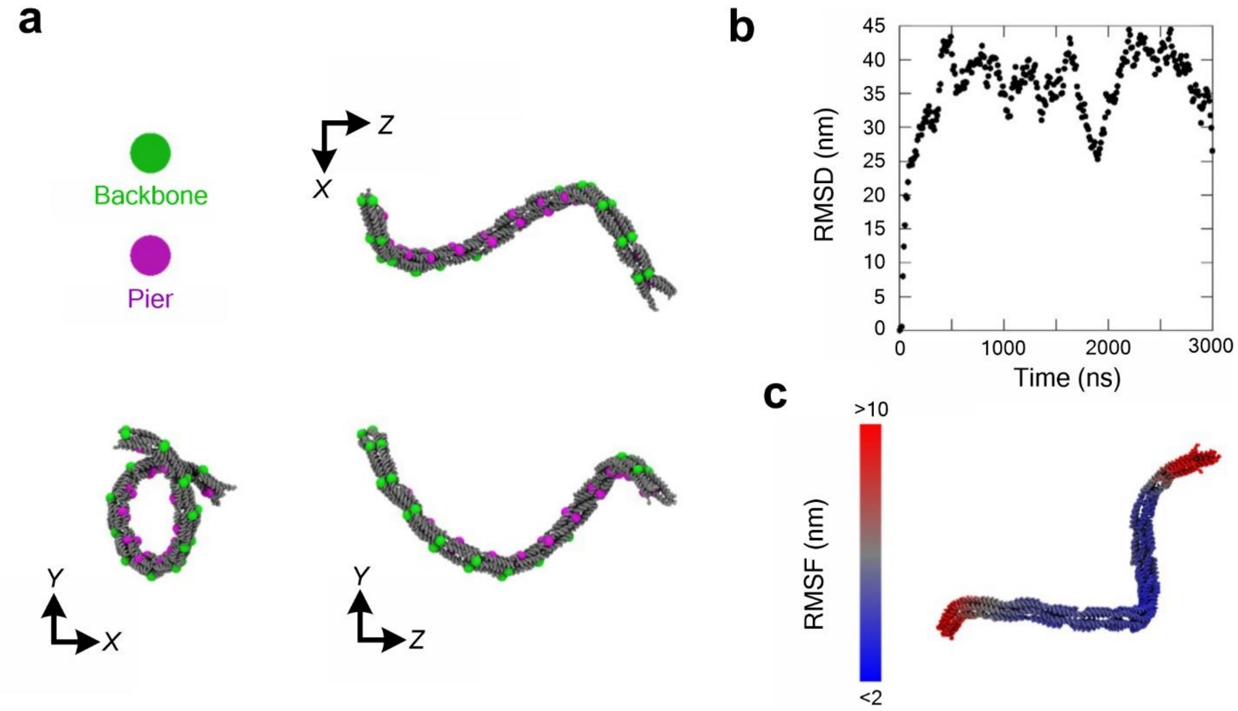

Suppl. Fig. 15: Detailed results of coarse-grained simulation where the 4 nt linkers were introduced as bridges. a Structure of the final frame at 3000 ns from orthographic views. Entities in backbone and pier are selected repeatedly and emphasized as green and magenta spheres, respectively. **b** Graph that shows the trajectory of RMSD **c** Graph that shows the RMSF in blue-to-red color scale, which is computed from 100 structures between 2000 to 3000 ns.

Suppl. Fig. 16. Simulation results fitted to form helix showing radius and pitch

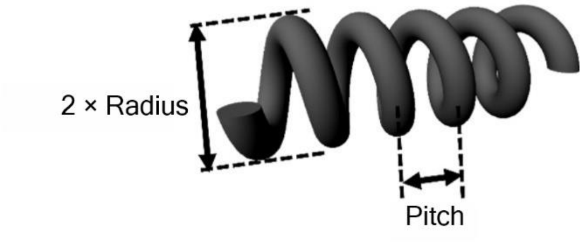

Suppl. Fig. 16: Specification of the radius and pitch of a helix. Radius of a helix corresponds to the radius of the circle which is formed when the helix is projected to a plane perpendicular to the axis of the helix. The pitch of the helix corresponds to the distance between the repetitive period when the helix is projected to a plane parallel to the axis of the helix. (see Suppl. Notes for details).

Suppl. Fig. 17. Bar plots to show the thermal fluctuations have non-negligible effect on pitch of helix

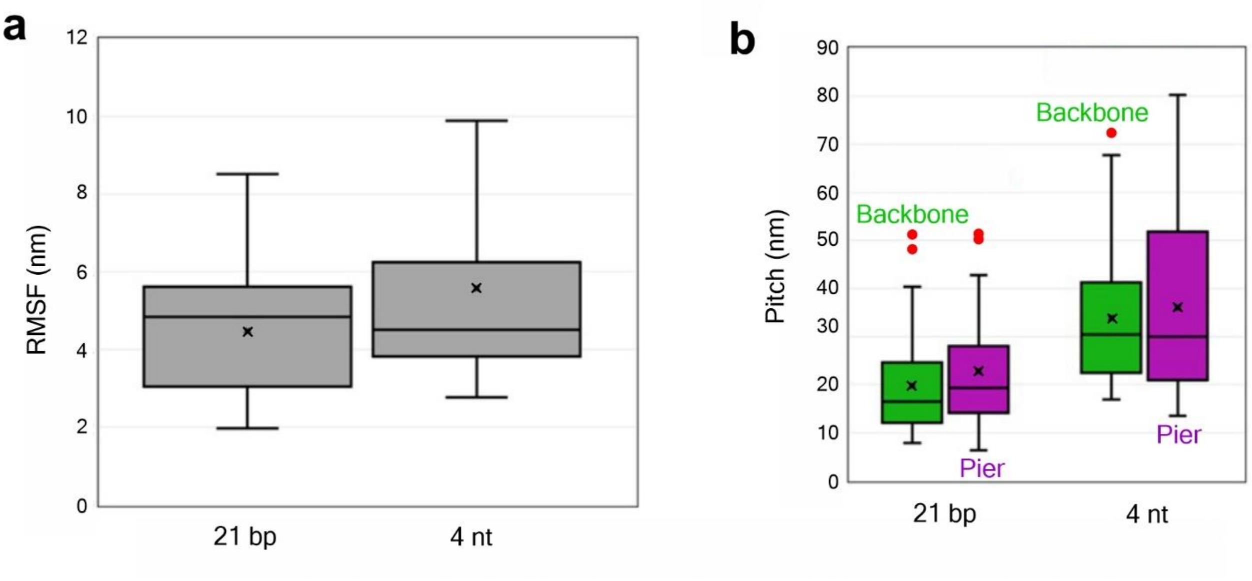

Suppl. Fig. 17: Distribution of the numerical values of the simulation results. a Root mean square fluctuations (RMSF) of all the entities in oxDNA simulation were computed using the last 100 frames in each linker, which show the effect of thermal fluctuations (n=13139 and 11315 for 21 bp and 4 nt cases, respectively). **b** Pitch of the fitted helix in the last 100 frames (n=100). In the graph, box represents the first and third quartiles, middle line shows the median, cross point is the mean, and bars are minimum and maximum while outliers are omitted in **a** and shown as points in **b**. In the case of the 21 bp linker, some entities with relatively large RMSF (*e.g.*, larger than 8 nm) make it difficult to fit the data points in some frames with relatively small pitches (*e.g.*, smaller than 8 nm). Red dots represent outliers. Since the number of measurements n is very huge, the mean value of the two distributions is statistically different by the t-test. The p value is almost zero.

Suppl. Fig. 18. Coarse-grained simulation with no linkers

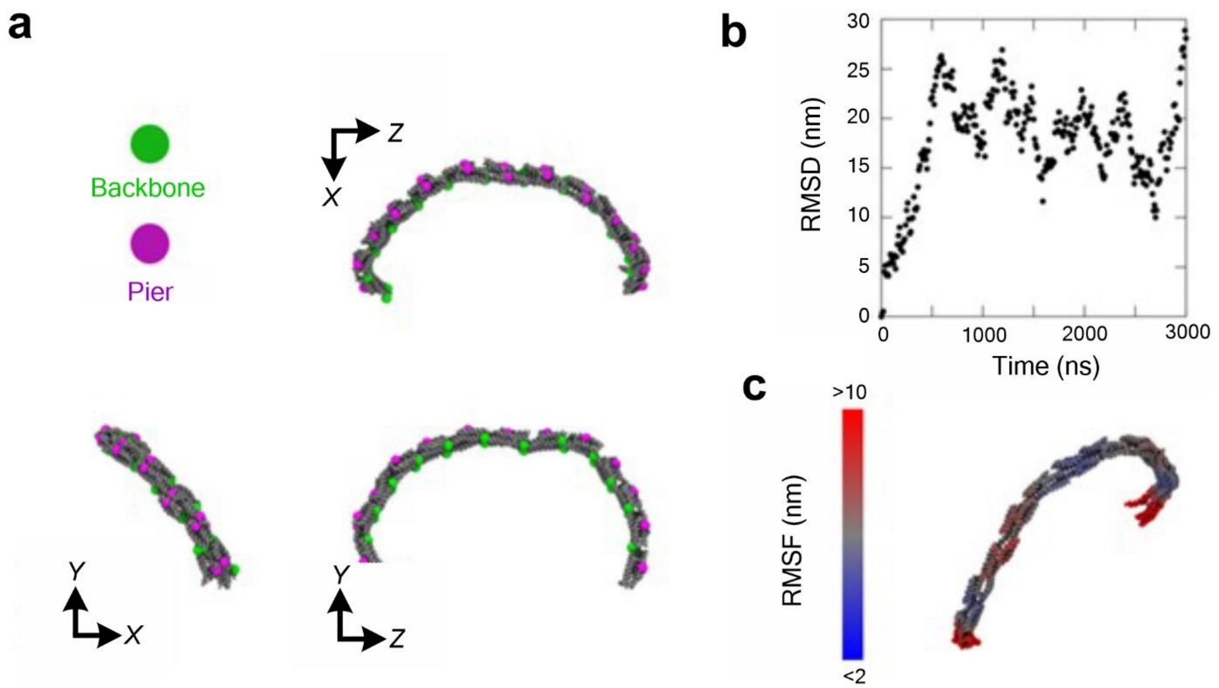

Suppl. Fig. 18: Detailed results of coarse-grained simulation where no linker was introduced as bridges. a Structure of the final frame at 3000 ns from orthographic views. Entities in backbone and pier are selected repeatedly and emphasized as green and magenta spheres, respectively. **b** Graph that shows the trajectory of RMSD **c** Graph that shows the RMSF in blue-to-red color scale, which is computed from 100 structures between 2000 to 3000 ns.

Suppl. Fig. 19. Coarse-grained simulation with single-stranded 21 nt linker

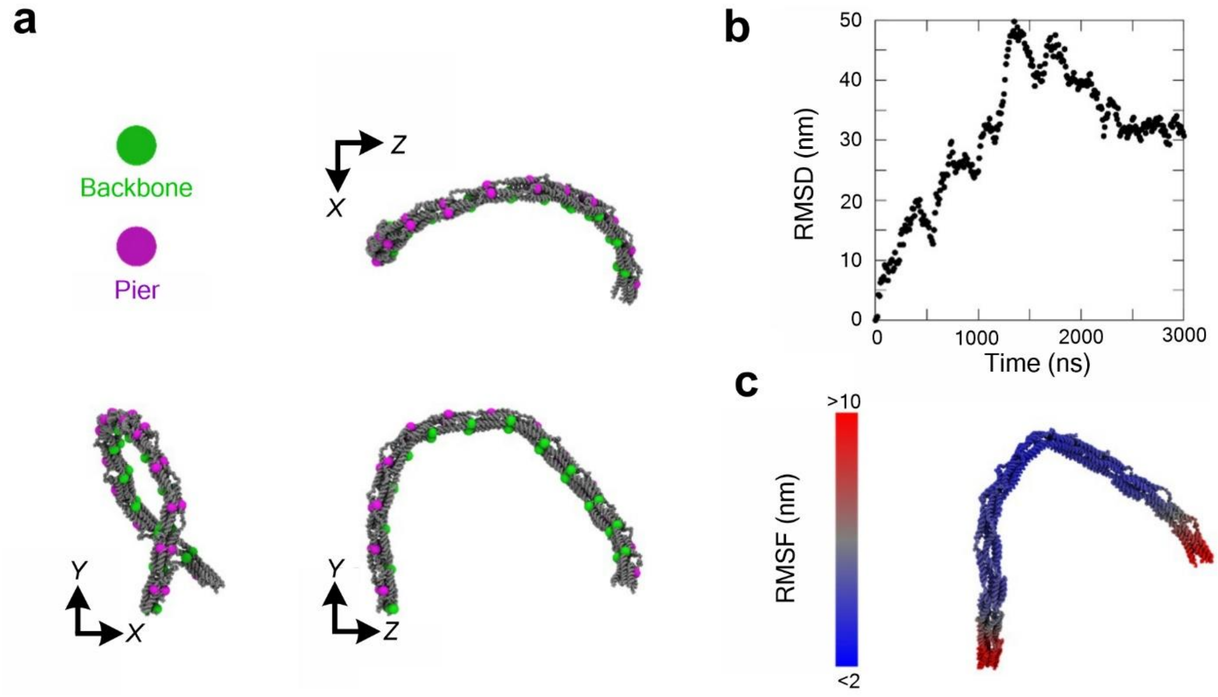

Suppl. Fig.19: Detailed results of coarse-grained simulation where single-stranded 21 nt linkers were introduced as bridges. a Structure of the final frame at 3000 ns from orthographic views. Entities in backbone and pier are selected repeatedly and emphasized as green and magenta spheres, respectively. **b** Graph that shows the trajectory of RMSD **c** Graph that shows the RMSF in blue-to-red color scale, which is computed from 100 structures between 2000 to 3000 ns.

Suppl. Note: Specification of the radius and pitch of a helix.

Using a parameter t, the equation of a helix is given as a trace of

$$x(t) = r\cos(t) \quad (1)$$

$$y(t) = r\cos(t) \quad (2)$$

$$z(t) = pt/2\pi, \quad (3)$$

where r is the radius and p is the pitch of the helix. Here, t is an auxiliary variable to simplify the mathematical representation. The data points of the simulation were fitted to the equation together with a matrix of translation and rotation.
